# Study on the Global Sustainability of the Korean Construction Industry Based on the GRI Standards

**DOI:** 10.3390/ijerph20054231

**Published:** 2023-02-27

**Authors:** Eunsoo Park, Younghyun Kim, Anyong Lee, Jieun Kim, Hyunseok Kong

**Affiliations:** 1 Department of Architecture, Sahmyook University, Seoul 01795, Republic of Korea; 2 Department of Construction Policy Research, Korea Institute of Civil Engineering and Building Technology, Goyang-si 10223, Republic of Korea; 3 Department of Smart Factory, Korea Polytechnics, Incheon 21417, Republic of Korea; 4 Department of Spatial Culture Design, Kookmin University, Seoul 02707, Republic of Korea; 5 College of Animal Biotechnology & Resource, Sahmyook University, Seoul 01795, Republic of Korea

**Keywords:** GRI Standards, ESG, sustainability management, global construction industry, sustainability, construction company

## Abstract

This study began with the increasing importance of ESG through sustainable management evaluation across all industries, predicting market demand through the ESG management paradigm and financial environment changes in the global industry, and establishing international strategies for the construction industry. Compared to other industries, the construction industry is in the early stages of ESG formation, and it is unclear how to expand its base by establishing evaluation system standards such as innovation of individual services, interaction of social capital, and definition of stakeholders. Currently, some large construction companies in the construction industry are publishing sustainability management reports at the group level, but given the recently strengthened global sustainability of ESG by GRI Standards, efficient analysis of global construction markets and strategic orders are needed. Therefore, this study focuses on assessing the sustainability strategies and directions of the construction industry from an ESG perspective. To this end, sustainability issues and insights, as well as global issues in Korea and the worldwide construction sector, were analyzed. The analysis showed that global construction companies were highly interested in business management approaches, such as safety and health, as critical issues regarding the construction industry’s sustainability strategy. In contrast, South Korean construction companies prioritize business values such as value creation, fair trade, and win-win. Both global and South Korean construction companies have been working on greenhouse gas reduction and energy sustainability. Regarding other issues, cultivating construction specialists, enhancing the job training system, and limiting serious accidents and safety mishaps were all significant from a social standpoint among South Korean construction companies. Conversely, global construction companies appeared to focus on issues related to ethical and environmental management from an organizational standpoint.

## 1. Introduction

As corporate uncertainty grows due to the coronavirus disease 2019 (COVID-19) pandemic that started in early 2020, the relevance of environmental, social, and governance (ESG), a non-financial issue, is emphasized for long-term management [1]. In this study, non-financial issues refer to various social impact values and indicators, not financial quantitative indicators that evaluate economic value such as corporate operating profit. ESG management is directly tied to corporate funding, which may result in capital market flows [2]. ESG management is no longer an option but a necessity and is growing as a significant indicator of corporate investment decisions alongside sustainable growth and corporate image improvement.

Various domestic and international institutions have proposed evaluation standards and metrics to evaluate Korean companies’ ESG levels. However, the compositions of evaluation metrics and techniques (e.g., point allocation and weighting of each item) are slightly different for each institution, and it is difficult to gain public access to the data. Recently, the Korean government announced the K-ESG Guidelines (December 2021) to address disparities in ESG evaluation requirements among institutions. Although the guideline presents standardized metrics for organizations to conduct ESG management, it is not differentiated by reflecting industry-specific characteristics. The management of general construction companies was only at the level of managing risks for ESG. This is causing a change in corporate management style for the unpredictable crisis ahead. The construction industry, in particular, has higher carbon emissions than other industries, and it requires more active ESG management than other industries in order to improve workplace safety accidents and governance.

The importance of ESG in terms of actual investment in construction companies is rapidly growing. The size of social responsibility investment in sustainable management is also increasing. If Korean construction companies meet ESG standards, it may be advantageous not only for overseas financing but also for entering overseas markets. In Korea, the case of overseas development projects is typical. For example, when supporting investment funds by development banks, the environmental and social responsibility standards of the Organization for Economic Cooperation and Development (OECD) and the International Finance Corporation (IFC) are used as major standards The Korean construction industry needs a strategic approach to sustainable management in the global construction market.

In particular, ESG ratings affect credit ratings in South Korea. When referring to ESG when reviewing credit ratings, credit rating organizations are also involved in corporate funding. As a result, Korean construction companies have adopted ESG as their management policy and redesigned their organizations and businesses to reflect this.

According to the Construction Company A survey [2], interest in ESG management has increased in the construction industry since the COVID-19 outbreak. Furthermore, as the importance of ESG management has been recognized, major construction companies are working on acquiring relevant competencies.

However, when construction companies’ size shrinks, preparing response measures for ESG management becomes more difficult. Consequently, it is necessary to create strategic construction sustainability activation plans, such as guidelines and incentives, to vitalize ESG management in the construction industry.

Based on the Sustainability Accounting Standards Board, Henriksson [3] suggested a plan to integrate and apply ESG evaluations to all organizations, including construction companies. Kotsantonis [4] examined the extent to which ESG reflection in management provides benefits by comparing the myths and realities of ESG. According to [5], ESG management reports are best for measuring corporate sustainability. However, there is a lack of transparency and consistency in the data sources, weightings, and procedures. Thus, they suggest that when investors use the ESG rating table, it is appropriate to use ESG evaluation metrics that match investors’ investment purposes. Existing research has primarily focused on using ESG metrics and their relationship to management initiatives.

In another research direction, Sokolov [6] used natural language processing to substitute textual information with ESG scores and proposed strategies to improve and standardize the ESG scoring technique. Zhang [7] investigated the extent to which ESG performance is considered in corporate loan assessments. According to Li [8], ESG is a critical framework for firms pursuing sustainable growth, but more detailed research and systematic ESG evaluation methods should be studied further.

As demonstrated in the preceding discussion, scholarly interest in ESG issues is growing rapidly. In South Korea, the important keywords of ESG research, identified as the top 10 in the past 10 years, have been ESG, society, corporate, responsibility, management, governance, investment, sustainability, corporate social responsibility (CSR), and environment [9]. Environmental and carbon challenges were increasingly mentioned in research in 2021. Academic interest is growing year after year, but the fact is that research is focused on accounting and finance, such as stocks, investments, and Morgan Stanley Capital International.

In South Korea, major construction companies publish sustainability reports at the group level. However, given the recently reinforced ESG global sustainability requirements following the Global Reporting Initiative (GRI) Standards, it is necessary to strategically approach the construction order market by efficiently analyzing the expansion of and global insights into the international construction market. It is vital to analyze sustainability management strategies and key ESG issues to ensure international-level industrial competitiveness in terms of ESG sustainability in the Korean construction sector.

Therefore, this study focuses on assessing the sustainability strategies and directions of the construction industry from an ESG perspective. To this end, sustainability issues and insights, as well as global issues in Korea and the worldwide construction sector, were analyzed.

The ESG sustainability reports of the top 10 major construction companies in terms of construction capacity evaluation in South Korea were utilized as sample data for this study, and data analysis was performed by focusing on materiality evaluation issues in the reports. The global construction sectors were examined for major construction corporations in the top 10 based on the *Engineering News-Record* (*ENR*), excluding those who had not yet submitted their ESG management reports. The subjects of this study are major construction companies in the private sector, excluding the public sector, with a focus on representative construction companies that significantly influence the construction market in terms of construction orders. The scope of the subjects was narrowed by focusing on the provision and utilization of information in their annual sustainability reports. The following are the main contents of this study:

− Understanding the concepts and characteristics of the construction industry, sustainability, and sustainability management standards and systems through the literature;
− Analysis of major issues and agendas in ESG materiality evaluation by South Korean and global construction companies;
− Classification of major issues and agendas according to GRI Standards;
− Deriving insights and relationships between sustainability and ESG in the construction industry, focusing on classified issues.

Through this study, we hope to explore future ESG sustainability issues in the South Korean and global construction industries and their expandability to global insight review data.

## 2. ESG and Sustainability Management in Construction Industry

### 2.1. ESG and Sustainability

ESG stands for “environmental, social, and governance”, which are three key elements of corporate management that focus on environmental management, social responsibility, and sound and transparent governance to achieve sustainable management [10].

“Sustainable development” first appeared in *Our Common Future*, published by the World Commission on Environment and Development in 1987, and was defined as “development that meets the needs of the present without compromising the ability of future generations to meet their own needs” [11]. Since then, the goals for sustainable development have been debated and established, and the United Nations (UN) General Assembly adopted the Sustainable Development Goals (SDGs) in 2015 [12]. In this social climate, a tendency has emerged for investors to invest in companies that have a positive social impact.

The triple bottom line (TBL), first proposed by Elkington in 1994, emerged to define CSR. The concept of TBL is that companies must balance financial, environmental, and social benefits. Major environmental accidents, such as the Bhopal gas tragedy (1984) at a chemical plant in India and the Exxon Valdez oil spill (1990) in the US, created an atmosphere in which the environment should be included in the scope of SRI’s social responsibility, which is not to invest in socially harmful contracts or companies. With the advent of TBL, the environment has entered the scope of social responsibility [13].

Subsequently, the Coalition for Environmentally Responsible Economies established the GRI with the premise that companies should take responsibility for environmental and social issues in management. In 1999, the United Nations Environment Program adopted the GRI as a global sustainability report guideline [13].

Governance was introduced as a regular component in the 2000s when it became clear that good governance increased business value. Later, as the word ESG was employed in the United Nations’ 2006 Principles for Responsible Investment, it came to mean sustainability management and three important pillars of corporate sustainability management. GRI Standards, ISO 26000, and the Global Compact are examples of sustainability evaluation metrics that encompass these characteristics and are utilized internationally, as shown in Table 1. The UN SDGs serve only as guidelines rather than as evaluation metrics.

The GRI Standard is the most representative indicator for evaluating a company’s non-financial factors. It was first established in 1999, and E, S, and G were all reflected and established in 2016.

### 2.2. GRI Standards and Evaluation of Global Materiality

The criticality evaluation method specified in the GRI Standard is as follows [14]: An organization is faced with a wide range of topics that it can report. Relevant topics that merit inclusion in the report are those that can reasonably be considered important for reflecting the organization’s economic, environmental, and social impacts or influencing the decisions of stakeholders. In this context, “impact” refers to an organization’s effect on the economy, environment, and/or society (positive or negative). A topic can be relevant, and thus potentially material, based on only one of these dimensions.

In sustainability reporting, materiality is concerned with two dimensions, a wider range of impacts and stakeholders. The principle determines which relevant topics are sufficiently important to be reported. Not all material topics are of equal importance, and the emphasis within a report is expected to reflect their relative priority.

A combination of internal and external factors can be considered when assessing whether a topic is material, as shown in Table 2. These include the organization’s overall mission and competitive strategy and the concerns expressed directly by stakeholders. Materiality can also be determined by broader societal expectations and by the organization’s influence on upstream entities, such as suppliers, or downstream entities, such as customers. Assessments of materiality are also expected to consider the expectations expressed in international standards and agreements with which the organization is expected to comply.

These internal and external factors must be considered when evaluating the importance of information to reflect significant economic, environmental, and/or social impacts or for stakeholders’ decision making. Various methodologies can be used to assess the significance of these effects. In general, “significant impacts” are a subject of established concern for expert communities or identified using established tools, such as impact assessment methodologies or life cycle assessments.

### 2.3. Sustainability Management and K-ESG of the Construction Industry

The UN SDGs, Global Compact 10, GRI, and ISO26000 are current global projects related to sustainable social values in the development cooperation process. They meticulously adhere to international standardization norms. At the World Economic Forum, it was agreed that the future of the construction industry, which has a significant impact on society, the economy, and the environment, will be shaped by several megatrends, including markets and customers, sustainability and resilience, society and labor force, and politics and regulation. Consequently, GRI Standards were actively implemented in the construction industry in July 2018.

The South Korean construction industry is also spreading sustainability management through CSR, creating shared value (CSV), and ESG activities, such as environmental friendliness and energy conservation, under the leadership of major construction companies, as shown in Table 3.

The importance of ESG began to emerge in South Korea around 2020. ESG is expected to be applied as a non-financial evaluation metric for enterprises in earnest, with the “K-ESG Guidelines” announced in December 2021. If the results of the materiality evaluation are not correctly reported during the K-ESG review, the company fails in the information disclosure part. This is similar to the failure of a corporation to disclose its financial status and impacts investment. This contrasts with CSR and CSV activities and disclosures, which have been largely left to corporate autonomy.

In particular, ESG is becoming increasingly important in evaluating sustainability management. Owing to changes in the management paradigm and financial environment, there is a need in the recent global industry to seek forecasting based on market demand and worldwide strategy establishment in the construction industry through ESG.

However, the construction industry is still in the early stages of ESG formation compared with other industries. Concerning ESG, it is unclear how to broaden the base through implementing assessment system criteria such as individual service innovation based on construction industry peculiarities, interaction with social capital, and stakeholder definition. Some large construction companies currently disclose sustainability reports at the group or company level. However, given the worldwide sustainability of ESG, as defined by the recently reinforced GRI Standards, it is vital to develop the international construction industry in the future, assess global insights efficiently, and approach the order market strategically.

To that end, this study examines sustainability management strategies and ESG-based core issues to ensure international-level industrial competitiveness in the South Korean construction industry regarding ESG sustainability.

## 3. Sustainability Analysis of the Construction Industry According to GRI Standards

### 3.1. Analysis Design

The ESG management direction of the South Korean construction industry [19,20,21,22,23,24,25,26] and the sustainability management directions of major overseas construction companies [27,28,29,30,31,32,33] were compared using materiality evaluation, which is an essential component of GRI evaluation. For sustainability management, materiality evaluation is a major issue that sets the direction for a company’s present and future sustainability management. Since some data in South Korea are labeled differently, such as CSR reports rather than sustainability reports in some institutions, only the disclosed ESG-based materiality evaluation and analysis results were gathered. Furthermore, because too many issues were addressed, only data identified as core issues resulting from materiality analysis were gathered. Different numbers of issues were collected from each company during this process. Korea was surveyed based on the top 10 companies in the construction capability evaluation standard announced by the Ministry of Land, Infrastructure and Transport. Korea’s construction capability evaluation ranks the representative companies with the most construction performance in Korea, which is announced every year by the Ministry of Land, Infrastructure and Transport, a government department.

In other countries, ESG materiality evaluations were included in integrated annual reports for all but 3 of the top 10 construction companies. For the global top 10 companies, *ENR*, a US weekly magazine that provides news, analysis, data, and opinions on the global construction industry, was selected as the reference material. Data on the core issues were collected and analyzed as a result of each construction company’s materiality evaluation. Table 4 presents the analysis data for South Korean and global construction companies.

Sustainability issues and insights of South Korea’s top construction companies were derived by assessing the frequency of materiality evaluation issues based on GRI Standards, as shown in Table 5. The GRI Standards are a non-financial area evaluation approach that serves as the foundation for ESG, and they were utilized to categorize issues derived from nine South Korean and seven international construction companies. Correlation analysis with the UN SDGs was conducted to examine the core issues of the construction industry from an ESG standpoint, and the collected data were also analyzed using R programming (version 4.2.2).

Several data analysis processes were used in this study to identify materiality evaluation issues according to the GRI Standards. Construction companies’ management reports identify the GRI standard items that correspond to the topics chosen for the evaluation of materiality. However, unlike other companies, South Korean companies are often vague. To remedy this issue, the GRI code was verified by comparing the page written on the report with the relevant information on the issue and the page written on the GRI index provided in the sustainability report. If this procedure did not resolve the issue, the final GRI code was determined by listing the tasks or business keywords supplied in the report and comparing them with the GRI codes of other construction companies discovered in the preceding method.

### 3.2. ESG Sustainability Issues of Construction Companies through GRI Standards

A materiality evaluation was conducted for nine major construction companies that provide sustainability reports among the top 10 construction companies in the capability evaluation ranking in South Korea. Through this process, ESG issues selected by each construction company as core issues were converted into GRI standard evaluation items and categorized. In the categorization process, the corresponding index codes were matched according to GRI Standards based on the top 10 rankings of issues derived through materiality evaluation for each construction company, as shown in Table 6. The issues identified for each construction company were then categorized as unified information. In the categorization process, if any issue had a comprehensive meaning and could not be matched individually, it was categorized into multiple GRI indices.

For global construction companies, the corresponding GRI items for each materiality evaluation issue were specified. Therefore, the related information was derived without a conversion process, as shown in Table 7.

The results of converting the core issues derived from each construction company through a categorization process according to GRI Standards are summarized in Table 8. The GRI standard items used until 2021 comprise 36 items in total, excluding 101, which refer to the GRI Standards. Of these, 34 GRI items were mentioned by the global construction companies, and 29 were mentioned by the South Korean construction companies. Neither South Korean nor global companies mention Market Presence (202) or Public Policy (415). Item 202 refers to the market situation, but the details are about the employment rate and employment equality in the community in which the company is located. The way they interact with the community remains beneficial. Item 415 overlaps with Public Policy (103). It was found that the overseas construction company did not mention 415, even though they created a separate internal carbon policy.

The South Korean companies did not mention Tax (207); Labor/Management Relations (402); Freedom of Association and Collective Bargaining (407); Security Practices (410); Rights of Indigenous Peoples (411); five items, including 207, related to corporate tax transparency; or four items related to community.

### 3.3. Comparison of Sustainability Issues between Global and South Korean Construction Companies

**Figure 1:**
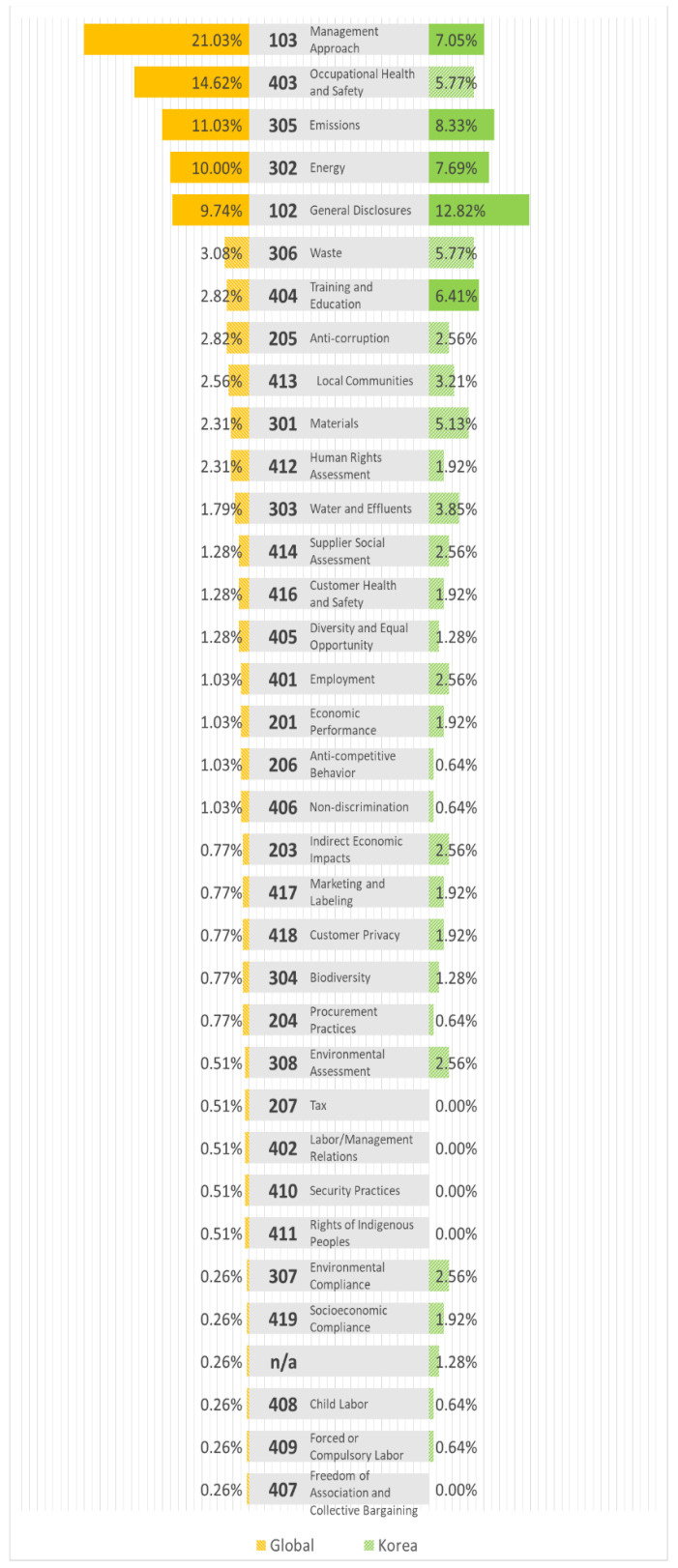
Frequencies of GRI items compared between global and South Korean construction companies.

The results of the analysis of the frequencies of GRI codes corresponding to each major issue in South Korea and overseas are shown in Figure 1. The issues in the top five ranks, including items with the same frequency, are marked in bold. The listing is in the order of overseas frequency ranking. Items with 6% or higher are highlighted in bold.

Table 9 outlines the top five global issues with a significantly different frequency than the other items and GRI items with a frequency of at least 6% in South Korea. Overseas construction companies value 103 and 403 highly, whereas South Korean construction companies value 102 in the same ESG group and assign higher importance to 305 and 302, which correspond to the environment, with 102. The list is ordered by frequency among the overseas companies.

In addition to analyzing the details according to the GRI Standards, the contents of the core issues are analyzed in Table 10 and Table 11. The keywords of the 300s are identical because the aim to be attained by the comprehensive project by 2050 coincides with the existing desires of the company’s external stakeholders. The response to the climate crisis is regarded as the world’s most pressing concern, which explains why new eco-friendly technology keywords stand out in the management direction of companies.

In South Korea, the Serious Accidents Punishment Act’s social expectations have not been adequately reflected in business management in a timely manner. In contrast, item 403, related to the safety of corporate executives and employees, is a key issue for global companies. Moreover, item 103, related to corporate management direction, was also dealt with as a critical issue to the extent that a term in 103 was selected as a keyword.

### 3.4. Correlation between Sustainability and ESG in Construction Industry

The current situation of the core sustainability issues of global and South Korean construction companies varied by ESG categorization, as shown in Table 12. According to this table, both South Korean and global companies are interested in the three ESG issues. They have a significant interest in the environment (E) domain, including detailed information related to item 103. This is attributable to the climate crisis response goals of 2030 and 2050. It was discovered that in the community (S) sector, there was a greater interest in social values within the company than in carrying out social responsibility activities outside the company or creating shared values. Global companies are more concerned with safety and health, whereas South Korean companies are strongly interested in enhancing their employees’ abilities.

## 4. Discussion

The sustainability of the construction industry was analyzed based on the materiality evaluation of the ESG-based sustainability reports of construction companies. Consequently, the following academic and social insights were derived as sustainability strategies for the construction industry.

Reducing greenhouse gas emissions from environmental sources is a core issue in the construction industry. The response to climate change has emerged as one of the world’s most pressing issues. Major governments worldwide proclaimed Net Zero by 2050 in 2020, emphasizing the importance of cities as places where policies meet people to achieve carbon neutrality.

As a detailed business strategy, it is necessary to promote a plan to advance the emission management system by improving energy efficiency in the workplaces of construction companies and a strategy to expand new eco-friendly businesses through the development of new technologies and product services. Rebuild is a representative example of an overseas construction company’s zero-energy and social innovation activities. Rebuild bases its renovation ecosystem on the integration of profitable technologies, business models, and the interaction of the life cycle with different types of residential renovations. This innovation creates a multiple-collaboration framework within a rehabilitation methodology managed by an agile project management tool capable of interconnecting the key steps of a custom-made renovation plan in real time between all agents involved in the value chain of building rehabilitation.

South Korea has also established a national goal of reducing emissions by 24.4% (relative to 2017) by 2030 to achieve carbon neutrality by 2050, following the Paris Agreement. South Korea’s key carbon-neutral policies are centered on the source of emissions, and limitations in sectoral methods may persist, necessitating the strengthening of the carbon-neutral policy promotion system at the national and city levels (Korea Research Institute for Human Settlements, 2021).

It is necessary to consider developing an active strategy to address the global climate crisis, such as developing a mid- to long-term emission target-setting plan at the construction industry level using the Task Force on Climate-related Financial Disclosures standard and the Science Based Targets Initiative following international standards.

Along with efforts to reduce greenhouse gas emissions, the public interest is growing in expanding renewable energy and eco-friendly industries. The construction industry is also hastening the development and conversion of an environmentally friendly industrial infrastructure. Construction companies are partnering with the government’s energy strategy and the international community to cut carbon dioxide emissions to address the global warming challenge. They are focusing on growing their technological skills in environmentally friendly new and renewable energy businesses, such as geothermal power generation, solar power generation, and biomass power generation, as well as renewable energy total solution companies.

The wastewater and waste sectors have focused on developing solutions to improve resource efficiency. New technologies for fueling and reusing wastewater sludge are being developed. Construction material utilization technology, byproduct hydrogen recycling technology, water treatment convergence technology, and the construction of hydrogen production plants are all being developed. The construction industry must develop a strategy to activate the circular economy of resources by utilizing resources more efficiently and increasing the recycling rate of used resources.

In the spheres of safety and health, efforts can be made to reduce serious accidents and safety mishaps, which have recently become a problem in South Korea. Accidents can occur across facilities and service infrastructure because of the industrial nature of the construction industry. To this end, project stakeholders must define and implement uniform safety and health policies and goals. South Korea is bolstering the building of safety and health management systems, including identifying basic risk factors, elimination measures, and construction process control measures. South Korea has enhanced its legal requirements such that if a significant industrial accident occurs because of a failure to establish or execute a safety and health management system, the person responsible is punishable with more than one year in prison or a fine not exceeding KRW 1 billion.

The management approach is ethical management and internalization of compliance from an ethical point of view. Ethical corporate practices, fair trade compliance, and trust building should be extensively developed to implement societal values. Furthermore, company-wide efforts should be made to build a management culture with stakeholders, from business partners and subcontractors to business partners. The sustainability strategy for the construction industry is shown in Table 13.

Finally, as presented in Table 14, we compared the top five global and South Korean keywords. Win-win growth between global companies’ subcontractors and South Korean companies entails developing a culture of mutual growth with subcontractors based on fair trade and risk management. The main difference between South Korea and other countries regarding items 102 and 103 is employee issues. In South Korea, the most important issue was 404 (training and education), whereas, in other countries, the key issue was 403 (occupational safety and health). Accordingly, the safety, health, and rights of executives and employees are frequently addressed in corporate status (102) and management direction (103) in other countries. By contrast, value creation and business growth have appeared as significant keywords in South Korean companies because they are interested in developing their employees. In the management direction, climate crisis response appeared as a keyword because the climate crisis response goals of 2030 and 2050 are core issues.

Both global and South Korean construction companies appear to be carrying out projects in the same direction to reach the environmental pollution reduction target that they wish to attain nationally in the context of the climate crisis. However, while global companies are interested in conserving and utilizing energy, South Korea is more interested in developing new technology.

## 5. Conclusions

In addition to CSV, corporate management has recently strengthened sustainability management through ESG activities. Thus, interest in sustainability management in the construction industry is high. The sustainability state of the top 10 construction companies was examined in this study using the materiality evaluation results based on the GRI Standards during ESG operations in terms of sustainability management in the South Korean construction industry. We investigated improvement plans and strategies to achieve global sustainability in the construction industry by comparing core issues with global construction companies.

Sustainable management in the Korean construction industry is still in its early stages. Systematic management and operation and approach to ESG across the construction industry need to be attempted. This can be an important factor in securing corporate sustainability and competitiveness in the global market with a simple management strategy. In addition, this study is meaningful in comparing sustainability management with global construction companies and comparing the standards and values of sustainability pursued by the global construction industry.

To examine sustainability management in the construction industry, we examined the sustainability reports of South Korea’s top 10 major construction companies and *ENR*-based global construction companies. The sustainability of the construction industry was analyzed based on the key issues for each company derived from the ESG materiality evaluation data of each company report based on the GRI Standards.

The analysis showed that global construction companies were highly interested in business management approaches, such as safety and health, as critical issues regarding the construction industry’s sustainability strategy. In contrast, South Korean construction companies prioritize business values such as value creation, fair trade, and win-win. Both global and South Korean construction companies have been working on greenhouse gas reduction and energy sustainability. Regarding other issues, cultivating construction specialists, enhancing the job training system, and limiting serious accidents and safety mishaps were all significant from a social standpoint among South Korean construction companies. Conversely, global construction companies appeared to focus on issues related to ethical and environmental management from an organizational standpoint.

In particular, it was discovered that as an important issue in the sustainable construction industry, a strategic orientation in terms of environment, social, and governance from an ESG perspective is necessary. Given the future environmental characteristics of the construction industry, it is predicted that sustainability management and the formation of construction companies’ business strategies will be critical. Construction companies must focus on adopting strategies based on strategic communication and stakeholder participation in ESG sustainability management as a result of this.

However, the results revealed that South Korean construction companies did not systematically establish sustainable management strategies. Some South Korean construction companies did not indicate a link between core issues, such as materiality evaluation, and global sustainability metrics, such as GRI Standards. Therefore, a strategic approach for ultimate global ESG needs to be supplemented. Furthermore, in a survey of the top 30 construction companies, almost half of the companies indicated sustainability management on their websites, whereas the majority of major construction companies did not include sustainability initiatives. Furthermore, while most major South Korean construction companies recognize the necessity of ESG management, their responses still focus on domestic evaluation agencies; thus, there is a problem with not meeting the ESG strategy according to the overseas level. Considering the period when the South Korean government adopted K-ESG, there was no systematic strategy in the construction industry, unlike ESG activation in other industries. Therefore, an institutional supplementation system is required for this industry.

Finally, this study analyzed and confirmed the correlation of sustainability management issues between the top 10 major global and South Korean construction companies. In addition, in the case of global construction companies, only seven construction companies were investigated because some information was not disclosed for three companies. In the case of Korea, the size of the construction industry is variously distributed, and the market is widely formed. Therefore, there are limitations in evaluating the Korean construction industry with only a few major construction companies. However, due to the small sample size, there are limits to expanding the interpretation of these research findings to general phenomena in South Korean and global construction industries. Consequently, it is essential to subdivide the sample target companies’ data categorization systems and undertake additional verification and assessment in the future.

In addition, additional research and development on how it would be desirable to link the implementation of SDGs to management strategies in the process of promoting sustainable management by domestic construction companies is needed.

## Figures and Tables

**Table 1:** Standards and metrics of international organizations related to sustainability.

Standards	Supervisors	Details
GRI Standards	UN (United Nations)	- Corporate sustainability reporting guidelines for the economic, environmental, and social performance of companies - Evaluation is performed largely in four areas: general standards (GRI 100), economy (GRI 200), environment (GRI 300), and society (GRI 400)
UN SDGs	UN (United Nations)	- Goals that the international community must achieve to solve the global poverty problem and realize sustainability development - Composed of 17 goals and 169 targets
Global Compact	UN (United Nations)	- Emphasizes corporate transparency and social responsibility - Presents 10 principles, including human rights, labor, and environment
ISO 26000	ISO (International Organization for Standardization)	- Guidelines for Socially Responsible Management: Social Responsibility (SR) guidelines apply to all organizations, including businesses, government agencies, labor unions, and civic groups. - Evaluates core subjects: environment, human rights, labor practices, organizational governance, fair operating practices, community involvement and development, and consumer issues
TCFD recommendation	TCFD (Task Force on Climate-Related Financial Disclosures)	- Recommendations - Composed of four elements: governance, strategy, risk management, metrics, and targets

**Table 2:** Step-by-step materiality evaluation analysis method.

Step		Detailed Analysis	Content of Analysis
1	Select material issues (analyze internal and external environments)	External environment analysis	Laws, regulations, international agreements, or voluntary agreements of strategic significance to the organization and its stakeholders
		Media analysis	Reasonably estimable economic, environmental, and/or social impacts (such as climate change, HIV-AIDS, or poverty) identified through sound investigation by people with recognized expertise or by expert bodies with recognized credentials
		Management strategy analysis	The main topics and future challenges for a sector, as identified by peers and competitors
		International metrics and standards review	Metrics such as GRI Standards
2	Prioritize	Stakeholders’ interests survey	The interests and expectations of stakeholders specifically invested in the organization, such as employees and shareholders Broader economic, social, and/or environmental interests and topics raised by stakeholders such as suppliers, local communities, vulnerable groups, and civil society workers who are not employees
		Business impact assessment	Key organizational values, policies, strategies, operational management systems, goals, and targets The core competencies of the organization and how they can contribute to sustainable development
3	Internal review	Insider review	Consequences for the organization related to its impacts on the economy, the environment, and/or society (for example, risks to its business model or reputation) Material topics are appropriately prioritized in the report

**Table 3:** Sustainability projects related to construction.

Performing Organizations	Project Name	Description
PJT OK	Share House WOOZOO 2013 [15]	Social housing is supplied through corporate share house business. Provision of various types of residential space at a lower price than general rental housing. Short-term contracts such as two months and six months are available, providing an alternative for those seeking short-term temporary housing. Furthermore, this project contributes to solving the housing problem by providing another option for increasing single-person households.
POSCO	POSCO Steel Village, 2014 [16]	A global activity to improve the poor living environment by building houses and bridges in poor areas overseas using steel materials and construction methods.
KT	GIGA Story, 2014 [17]	This activity started with “Giga Island No. 1” in Imjado, Shinan-gun, Jeollanam-do, to bridge the information gap and build wireless communication infrastructure, such as installing Internet networks and providing IT solutions in mountainous and island regions at home and abroad.
Hyundai E&C	H-CONTECH Overseas Technical Volunteer Group, 2017 [18]	Technical exchange-type volunteers are dispatched overseas to improve the village and school environments and hold technology exchange seminars.
Daewoo E&C	Habitat, 2018 [19]	A home repair volunteer group. Residential environment improvement activities such as wallpapering old houses, replacing flooring and sinks, and insulating work.

**Table 4:** Status of analysis data.

Classification	Analysis Information	
	South Korea [19,20,21,22,23,24,25,26]	Global [27,28,29,30,31,32,33]
Sample criteria	Top 10 companies based on construction capability evaluation (announced by the Ministry of Land, Infrastructure, and Transport in 2021)	Top 10 companies by *Engineering News-Record* (*ENR*): a US weekly magazine providing news, analysis, data, and opinion on the global construction industry
Sample target	Nine major construction companies in the top 10 (excluding companies that have not disclosed ESG management reports)	Seven major construction companies in the top 10 (excluding companies that have not disclosed ESG management reports (3 Chinese companies))
Collection period	16 November 2021∼1 December 2021 (12 weekdays)	7 February 2022~27 February 2022
Collected information	Sustainability reports of nine companies (Report period: January–December 2020) - Samsung C&T, 2021 - HYUNDAI ENGINEERING & CONSTRUCTION, 2021 - GS E&C, 2021 - POSCO, 2018 - DAEWOO E&C, 2021 - HYUNDAI ENGINEERING & CONSTRUCTION, 2021 - LOTTE E&C, 2021 - DL E&C, 2021 - SK ecoplant, 2020 ※ Some reports with different names were also included, such as Corporate Citizenship Report, CSR Report, and Integrated Report.	Integrated annual reports of seven companies - ACS, 2020 - Bouygues, 2020 - Ferrovial Corporación, S.A., 2021 - HOCHTIEF, 2020 - Skanska AB, 2020 - STRABAG SE, 2020 - VINCI, 2020 ※ Materiality evaluation that meets the GRI standard criteria is provided as a non-financial factor item along with financial statements in the integrated report.
Detailed information gathered from the reports	- 78 issues on materiality evaluation in the top 10 by company - Key ESG agenda for each company - Analysis results of the top 10 issues by company ※ Some issues in the 5th, 6th, 7th, and 9th ranks of some reports are included.	- 57 issues from the top 7 companies in materiality evaluation ※ Some issues in the 10th and 11th ranks in some reports are included.

**Table 5:** Issue analysis framework for sustainability materiality evaluation of the construction industry.

Analysis Process	Main Analysis Details
1. Analysis of issues about sustainability management materiality evaluation in the construction industry	Subject of analysis: materiality evaluation issues of each company - Recategorization of materiality evaluation issues according to the GRI Standards - Expert review of recategorized items (1st) - Frequency analysis of materiality evaluation issues - Review of core issue insights
2. Analysis of global core issues of sustainability in the construction industry	Subject of analysis: top 5 core issues that have been recategorized - Categorization of global issues based on UN SDGs - Analysis of issue contents of existing companies according to the UN SDGs categorized by item - Analysis of the characteristics of main contents for each of the top 10 items - Expert review of the analyzed contents (2nd)
3. Correlation between sustainability and ESG in the construction industry	Subject of analysis: top 5 core issues that have been recategorized - Categorization of core issues according to each standard for the environment, social, and governance of ESG - Expert review of the analyzed contents (3rd) - Frequency analysis of each categorization item - Review of correlations and insights for each ESG item

**Table 6:** An example of GRI conversion table for construction company A in South Korea.

Construction Company	Issue Ranking	ESG Issues of Construction Company A	GRI Index Matching Information for Conversion into Unified Information	GRI Standards
Construction Company A	1	Climate change response	Recycling	301. Materials
			New and renewable energy Energy efficiency improvement	302. Energy
			Water recycling	303. Water and Effluents
			Greenhouse gas reduction	305. Emissions
Construction Company A	2	Employee safety and health	Realization of zero severe disaster	403. Occupational Health and Safety
Construction Company A	3	Responsible supply chain management	Compliance with labor and human rights laws	408. Child Labor
			Human rights protection	409. Forced or Compulsory Labor
			Workplace human rights inspection	412. Human Rights Assessment
			Supplier human rights inspection	414. Supplier Social Assessment
Construction Company A	4	Securing new growth engines	New technology R&D and innovation	103. Management Approach
Construction Company A	5	Establish a good corporate culture for work	Reward activation	401. Employment
			Leadership coaching training	404. Training and Education
Construction Company A	6	Strengthen ethical management	Anti-corruption guide	205. Anti-Corruption
Construction Company A	7	Strengthen responsibility for products and services	Prevention of physical harm to customers	416. Customer Health and Safety
Construction Company A	8	Human resources development and fair performance compensation	Job performance evaluation and compensation Retirement and job change support	401. Employment
			Job competency improvement training programs	404. Training and Education
Construction Company A	9	Responsible board composition and operation	Independent board of directors Diversity of board members Professional board Disclosure of the composition of the board Disclosure of the operation of the board	102. General Disclosures
Construction Company A	10	Integrated management of financial and non-financial risks	Risk management by an expert committee or board of directors	102. General Disclosures

**Table 7:** GRI table for global construction company B.

Construction Company	Materiality Evaluation Issues of Construction Company B	GRI Standards
Construction Company B	Responsibility with local communities	103. Management Approach
		413. Local Communities
Construction Company B	Efficient management of resources	103. Management Approach
		301. Materials
		302. Energy
		303. Water and Effluents
		306. Waste
Construction Company B	Development and talent of diversity	103. Management Approach
		404. Training and Education
Construction Company B	Ethical and responsible companies	103. Management Approach
		205. Anti-corruption
		206. Anti-competitive Behavior
Construction Company B	The climate: A global concern responsible	305. Emissions
Construction Company B	Zero accidents objective	103. Management Approach
		403. Occupational Health and Safety
Construction Company B	Responsible supply chain	103. Management Approach
		204. Procurement Practices
		308. Supplier Environmental Assessment
		414. Supplier Social Assessment

**Table 8:** ESG sustainability issues of construction companies through GRI Standards.

GRI Standards								
Code. Item Name	Global	South Korea	Code. Item Name	Global	South Korea	Code. Item Name	Global	South Korea
102. General Disclosures	O	O	304. Biodiversity	O	O	408. Child Labor	O	O
103. Management Approach	O	O	305. Emissions	O	O	409. Forced or Compulsory Labor	O	O
201. Economic Performance	O	O	306. Waste	O	O	410. Security Practices	O	X
202. Market Presence	X	X	307. Environmental Compliance	O	O	411. Rights of Indigenous Peoples	O	X
203. Indirect Economic Impacts	O	O	308. Supplier Environmental Assessment	O	O	412. Human Rights Assessment	O	O
204. Procurement Practices	O	O	401. Employment	O	O	413. Local Communities	O	O
205. Anti-corruption	O	O	402. Labor/Management Relations	O	X	414. Supplier Social Assessment	O	O
206. Anti-competitive Behavior	O	O	403. Occupational Health and Safety	O	O	415. Public Policy	X	X
207. Tax	O	X	404. Training and Education	O	O	416. Customer Health and Safety	O	O
301. Materials	O	O	405. Diversity and Equal Opportunity	O	O	417. Marketing and Labeling	O	O
302. Energy	O	O	406. Non-discrimination	O	O	418. Customer Privacy	O	O
303. Water and Effluents	O	O	407. Freedom of Association and Collective Bargaining	O	X	419. Socioeconomic Compliance	O	O

**Table 9:** Details of GRI codes with top frequencies among global and South Korean construction companies.

GRI Code	GRI Content	Global Ranking (Frequency (%))	South Korean Ranking (Frequency (%))
103	Management Approach - Explanation of the material topic and its boundary - Management approach and its components - Evaluation of the management approach	1 (21.03)	4 (7.05)
403	Occupational Health and Safety - Occupational health and safety management system - Hazard identification, risk assessment, and incident investigation - Occupational health services - Worker participation, consultation, and communication on occupational health and safety - Worker training on occupational health and safety - Promotion of worker health - Prevention and mitigation of occupational health and safety impacts directly linked to business relationships - Workers covered by an occupational health and safety management system - Work-related injuries - Work-related ill health	2 (14.62)	(6)
305	Emissions - Direct (Scope 1) greenhouse gas (GHG) emissions - Energy indirect (Scope 2) GHG emissions - Other indirect (Scope 3) GHG emissions - GHG emission intensity - Reduction of GHG emissions - Emissions of ozone-depleting substances (ODSs) - Nitrogen oxides (NOX), sulfur oxides (SOX), and other significant air emissions	3 (11.03)	2 (8.33)
302	Energy - Energy consumption within the organization - Energy consumption outside of the organization - Energy intensity - Reduction of energy consumption - Reductions in energy requirements of products and services	4 (10)	3 (7.69)
102	General Disclosures - Organizational profile - Strategy - Ethics and integrity - Governance - Stakeholder engagement - Reporting practice	5 (9.74)	1 (12.82)
404	Training and Education - Average hours of training per year per employee - Programs for upgrading employee skills and transition assistance programs - Percentage of employees receiving regular performance and career development reviews	(7)	5 (6.41)

**Table 10:** Detailed issues and derivation of keywords from issues corresponding to the top 5 GRI codes among South Korean construction companies.

Ranking	GRI	Corporate Issues (Number of Appearances)	Core Contents (Keywords)
1	102 General Disclosures	Composition and operation of the responsible board of directors/Integrated management of financial and non-financial risks/Nurturing new growth businesses and discovering promising future businesses/Improving customer satisfaction and creating customer value/Improving business performance and creating economic value (2)/Product, service, and quality innovation/Promotion of ethical and compliance management and anti-corruption/Mutual growth and win-win cooperation with partners/Strengthening government policies/regulations and violation of laws/Community Climate change/Safety and health/Supply chain/Improvement of labor-management culture and working conditions/Sound governance/Establishment of fair trade/Mutual growth and win-win cooperation/Improvement of labor-management culture and working conditions/Establishment of fair competition and fair trade	Value creation Fair trade Win-win Business growth
2	305 Emissions	Response to climate change (3)/Promotion of eco-friendly management and reduction of environmental impact/Reduction in construction waste emissions/Demand for the introduction of eco-friendly technology and materials/Eco-friendly technology/Climate change (2)/Energy/Air pollution/Wastewater and waste/Development of eco-friendly technology	Emissions reduction Development of eco-friendly technology
3	302 Energy	Response to climate change/Fostering new growth businesses and discovering promising future businesses/Enhancing competitiveness in eco-friendly technology, design, and construction/Reduction in construction waste emissions/Requesting introduction of eco-friendly technologies and materials/Development of eco-friendly construction methods and renewable energy technologies/Climate change/Eco-friendly technologies/Energy/Reinforcement of technological capabilities (R&D)/Energy saving/Development of eco-friendly technology	New business New technology Eco-friendliness
4	103 Management Approach	Securing new growth engines/Strengthening government policies and regulations and violations/Product responsibility/Wastewater and waste/Eco-friendly technology/Community (2)/Climate change/Innovative technology/Personnel management/Response to climate change	Eco-friendly technology Climate change Community
5	404 Training and Education	Establishment of a good corporate culture for work/Human resources development and fair performance compensation (2)/Product, service, and quality innovation/Promotion of eco-friendly management and reduction of environmental impact/Intensifying competition for talent acquisition/Employee competency development/Personnel management/Improvement of labor-management culture and working conditions/Response to climate change	Fair Compensation Performance Human resources development

**Table 11:** Detailed issues and derivation of keywords from issues corresponding to the top 5 GRI codes among global construction companies.

Ranking	GRI	Corporate Issues (Number of Appearances)	Core Contents (Keywords)
1	103 Management Approach	Responsibility with local communities/Efficient management of resources/Development and talent of diversity/Ethical and responsible companies/Zero accidents objective/Responsible supply chain/Safety and health/Innovation management/Digital transformation/Sustainable cities and mobility/Talent attraction and training/Climate change/Energy environment/Business ethics/Circular economy/Natural environments/Quality of customer and user experience/Client satisfaction/Digitalization and innovation/Occupational safety/Health protection/Strategic human resource development/Fair competition/Materials/Waste and circularity/Energy and emissions/Employment/Climate change: Adaptation/Diversity, inclusiveness, and non-discrimination/Customer and user satisfaction/Health, safety, and wellbeing of employees and contractors/Career development, diversity, and inclusion in the workplace	Safety Health Development Climate change Diversity
2	403 Occupational Health and Safety	Zero accidents objective/Safety and health/Health, safety, and security of employees, temporary staff, and subcontractors/Health and safety, and quality of life at work/Occupational safety/Occupational health and safety	Safety Health Occupational
3	305 Emissions	The climate: a global concern responsible/Climate change/Climate risk/New uses and adaptability of business models/Energy and emissions Climate change: GHG emissions/Climate change: Adaptation/Climate change	New energy Greenhouse gas (GHG) reduction Climate change
4	302 Energy	Efficient management of resources/Energy environment/Climate change/Climate risk/Innovation capacity/New uses and adaptability of business models/Energy and emissions/Energy/Climate change: Adaptation/Climate change	Climate change Energy conservation Energy-efficient
5	102 General Disclosures	Health, safety, and security of employees, temporary staff, and subcontractors/Employability and skills development/Human rights/Business ethics/Relations with suppliers and subcontractors/The group’s socio-economic footprint in regions (socio-economic contribution to regions)/Business ethics, respect for human rights, and compliance/Quality of customer and user experience/Innovation capacity/Innovation applied to business/Customer and user satisfaction/Health, safety, and wellbeing of employees and contractors/Climate change/Good corporate governance/Ethical behavior	For employees Subcontractors Human rights Ethics Customer Management innovation

**Table 12:** ESG categorization of core issues among global and Korean construction companies.

Global Construction Companies				South Korean Construction Companies			
Frequency Ranking	GRI Code	GRI Item	ESG	Frequency Ranking	GRI Code	GRI Item	ESG
1	103	Management Approach	G	1	102	General Disclosures	G
2	403	Occupational health services	S	2	305	Emissions	E
3	305	Emissions	E	3	302	Energy	E
4	302	Energy	E	4	103	Management Approach	G
5	102	General Disclosures	G	5	404	Training and Education	S

**Table 13:** Sustainability strategies for the construction industry.

Sustainability Strategy	Main Issue
Reducing greenhouse gas emissions from environmental sources	- Response to climate change - Net Zero by 2050 - Influence of Carbon Reduction Policy - Advanced emission management from environmental sources
Renewable energy and eco-friendly industries	- Geothermal power generation - Solar power generation - Biomass power generation - Renewable energy total solution
Wastewater and waste	- Fueling and reusing wastewater sludge - Construction material utilization technology - Byproduct hydrogen recycling technology - Water treatment convergence technology - Hydrogen production plants
Safety and health	- Reduction of serious accidents and safety mishaps - Strengthening the safety and health management system - Prevention of Facility and Service Infrastructure Accidents - Establishing a consistent health and safety policy
Management approach	- Ethical management and internalization of compliance - Fair trade compliance - Trust building - Enterprise stakeholder management

**Table 14:** Comparison of common keywords in four GRI codes corresponding to the top 5 between global and South Korean companies.

GRI	Global	South Korea
102	Subcontractors For employees Human rights Ethics Customer Management innovation	Win-win Value creation Fair trade Business growth
103	Development Climate change Safety Health Diversity	Eco-friendly technology Climate change Community
302	Climate change Energy conservation Energy-efficient	Eco-friendly New business New technology
305	New energy Climate change Responsible Adaptability Greenhouse gas (GHG) reduction	Eco-friendly technology development Emissions reduction
